# Legume Consumption Improves Cellular Health and Autonomic Function in Competitive Swimmers

**DOI:** 10.3390/nu18020274

**Published:** 2026-01-14

**Authors:** Elisabetta Camajani, Valerio Caporali, Stefania Gorini, Alessandra Feraco, Chiara Quattrini, Luigi Procaccio, Andrea Armani, Elvira Padua, Massimiliano Caprio, Mauro Lombardo

**Affiliations:** 1 Department for the Promotion of Human Sciences and Quality of Life, San Raffaele Open University, 00166 Roma, Italy; elisabetta.camajani@uniroma5.it (E.C.); valerio.caporali@studenti.uniroma5.it (V.C.); luigi.procaccio@gmail.com (L.P.); andrea.armani@uniroma5.it (A.A.); elvira.padua@uniroma5.it (E.P.); massimiliano.caprio@uniroma5.it (M.C.); mauro.lombardo@uniroma5.it (M.L.); 2 Laboratory of Cardiovascular Endocrinology, IRCCS San Raffaele, 00166 Roma, Italy

**Keywords:** Mediterranean diet, athletes, physical exercise, body composition

## Abstract

**Objective:** This study evaluated whether higher adherence to the Mediterranean Diet (MD), specifically through increased legume consumption, is associated with improved functional, autonomic, and performance parameters in adolescents and young adult competitive swimmers. **Methods**: Thirty-nine swimmers (mean age 19.7  ±  2.3 years; 22 men, 17 women) monitored over a five-month period under standardized training conditions. Based on baseline dietary assessment, participants were allocated into three groups according to habitual legume intake: Control group (<1 serving/week, no dietary modification), 3Legumes group (~2 servings/week, increased to 3/week), and 6Legumes group (~3–4 servings/week, increased to 6/week). Functional evaluation encompassed bioelectrical impedance parameters (phase angle, extracellular and intracellular water, ECW/ICW ratio), heart rate variability (HRV), cardiac coherence, and critical swimming speed test (CSS) results. **Results**: After 5 months, the 6Legumes group showed an increase in phase angle (Δ  =  +0.34  ±  0.35°, *p*  =  0.004), a reduction in extracellular water (Δ  =  −1.77  ±  0.93%, *p*  <  0.001), and an increase in intracellular water (Δ  =  +1.77  ±  0.93%, *p*  <  0.001), resulting in a lower ECW/ICW ratio (Δ  =  −0.051  ±  0.028, *p*  <  0.001). HRV (Δ  =  +6.92  ±  5.02, *p*  =  0.0003) and cardiac coherence (Δ  =  +0.40  ±  0.35, *p*  =  0.0015) also demonstrated statistically significant improvements, whereas CSS exhibited a positive trend (Δ  =  +0.011  ±  0.019 m/s, *p*  =  0.067) without reaching statistical significance. Between-group comparisons confirmed significant differences in phase angle and water-distribution parameters (all *p*  <  0.01). **Conclusions**: In this cohort of adolescents and young adult competitive swimmers, increased legume consumption within a Mediterranean dietary framework was associated with beneficial adaptations in cellular hydration status, autonomic regulation, and functional performance.

## 1. Introduction

An athlete’s diet must integrate the principles of both healthy eating and environmental sustainability, while providing sufficient energy and macronutrients to support optimal athletic performance [1,2]. The establishment of healthy dietary habits during adolescence is particularly crucial, as this period typically coincides with peak training loads and increasing nutritional requirements [3,4]. Appropriate nutrition for adolescents and young adults is essential to sustain growth, facilitate health maintenance, and optimize performance [5,6].

Transitioning globally to healthy diets by 2050 will necessitate substantial changes in dietary habits.

Healthy dietary patterns are characterized by adequate energy, emphasising plant-based foods, limiting animal-derived products, favoring unsaturated over saturated fats and minimizing refined grains, ultra-processed foods and added sugars. Within this framework, the EAT-Lancet Commission and other international recommendations, including sports nutrition guidelines, converge in calling for a global shift towards diets with less red and processed meat and added sugars and greater consumption of legumes, nuts, fruits and vegetables, in order to promote both human health and environmental sustainability [7,8,9,10,11,12].

The Mediterranean Diet (MD) stands out as a prototypical healthy dietary pattern, characterised by abundant plant foods alongside moderate amounts of lean animal proteins and olive oil, and is consistently linked to improved cardiometabolic, inflammatory and functional outcomes, including among physically active individuals [13,14]. The MD is closely aligned with current sports nutrition guidelines, which recommend high consumption of whole grains, legumes, fruits, and vegetables, complemented by moderate intakes of lean meats and healthy fats, providing a balanced variety of carbohydrate-rich foods that contribute to sustained energy availability, improved endurance, and effective post-exercise recovery [14,15]. Legumes represent a central component of the Mediterranean dietary pattern and provide a distinctive combination of high-quality plant protein, slowly digestible carbohydrates, soluble and insoluble fiber, and a wide spectrum of micronutrients, including potassium, magnesium and folate, together with bioactive polyphenols endowed with antioxidant and anti-inflammatory properties [16]. This nutrient profile is biologically relevant for cellular integrity and hydration because adequate potassium and magnesium intake supports intracellular electrolyte balance, while dietary antioxidants and polyphenols can mitigate oxidative and inflammatory insults that impair cell membranes and bioelectrical properties [16,17]. Consistent with this concept, recent observational data indicate that higher diet quality and greater consumption of minimally processed, plant-based foods are associated with more favorable phase angle values, suggesting a positive impact of plant-rich dietary patterns on cellular health [17,18]. Beyond cellular integrity, plant-rich diets have also been linked to improved autonomic regulation. Several studies in both general and specific populations report that higher intakes of fruits, vegetables and legumes, or adherence to vegetarian and Mediterranean-style dietary patterns are associated with higher heart rate variability and a more favorable sympathovagal balance, supporting a role for diet quality in modulating cardiac autonomic function [19,20,21]. In athletes, the MD has been proposed to enhance performance and recovery through its content of complex carbohydrates, unsaturated fats and polyphenol-rich plant foods, which may improve vascular function, oxidative stress defenses and systemic inflammation, thereby contributing to better endurance capacity and neuromuscular efficiency [15,22]. Within this framework, legumes are an attractive target for nutritional interventions in athletes because their dense matrix of plant protein, fiber, minerals and polyphenols may favorably influence phase angle, body water distribution and autonomic nervous system balance, potentially translating into measurable benefits for functional status and sport performance [15]. In order to address this hypothesis in the population under investigation, we therefore selected bioelectrical-impedance-derived phase angle and hydration indices as the primary endpoints of the study. Phase angle derived from bioelectrical impedance analysis (BIA) is widely regarded as an integrated marker of “cellular health” because it reflects the balance between resistance and reactance, thereby capturing cell membrane integrity, cellular mass and fluid distribution [23,24,25]. Higher phase angle values have been consistently associated with superior muscle quality, better functional status and more favourable clinical outcomes across diverse populations, including athletes, which supports its use as a validated, non-invasive indicator of cellular integrity and nutritional status [26,27,28]. In addition, BIA-derived hydration metrics such as extracellular and intracellular water and their ratio (ECW/ICW) provide insight into fluid compartmentalization rather than total body water alone [25,29]. A relative expansion of extracellular water and an increased ECW/ICW ratio are typically interpreted as markers of fluid shift, low-grade inflammation or compromised cellular integrity, whereas a larger intracellular water compartment is characteristic of well-hydrated, glycogen-replete muscle tissue [29,30]. In athletes, dynamic changes in ECW, ICW and ECW/ICW have been related to nutrition and hydration strategies, training load and tapering, and have been proposed as objective indicators of recovery status and readiness that complement traditional performance measures [31,32]. Summarizing, the Mediterranean dietary pattern represents an optimal nutritional framework for young athletes, provided that individual energy requirements, adequate hydration and recovery, are appropriately addressed. Recent evaluations of plant protein quality indicate that, when appropriately selected and combined, plant-derived proteins can provide adequate amounts of indispensable amino acids to support health and physical function, including in active individuals [33,34].

Despite these advantages [16,17], a persistent misconception remains that plant-based proteins are less effective than animal-derived proteins for supporting athletic performance and muscle recovery. This assumption may dissuade athletes from increasing their intake of legumes and other plant protein sources.

In fact, although interest in plant-based and Mediterranean-style dietary patterns in athletes is increasing, most studies have focused on overall diet quality rather than trying to highlight the effects of specific plant protein sources [22,35]. In particular, there is a paucity of data on the impact of legumes per se, within a Mediterranean diet framework, on bioelectrical-impedance-derived indices of cellular integrity and hydration or on autonomic measures, such as heart rate variability in competitive athletes, and the limited evidence linking discrete dietary components to HRV and BIA metrics remains largely observational and rarely sport-specific [28,36,37]. These gaps highlight the need for targeted interventional studies evaluating whether increasing specific plant-derived protein intake, such as legume consumption in the context of an MD, can elicit measurable adaptations in bioelectrical markers of cellular health, fluid distribution and autonomic regulation in competitive athletes. There is a clear need for sport-specific, mechanistic studies within a Mediterranean diet framework. Accordingly, the present study was designed to investigate whether increasing legume consumption in competitive swimmers, without altering training load or other major dietary components, is associated with favourable adaptations in phase angle, body water compartmentalisation and indices of autonomic regulation. We hypothesised that higher legume intake would improve cellular integrity, reflected by increased phase angle and a more favourable ECW/ICW ratio, and enhance autonomic balance, as indicated by higher heart rate variability and cardiac coherence over the course of a competitive season [22,35,36,38]. To address these issue, we conducted an intervention study involving adolescent and young adult competitive swimmers to determine whether increased adherence to the Mediterranean Diet—particularly through increase in legume consumption—was associated with improvements in functional and physiological outcomes throughout a competitive season. We selected competitive swimmers as the study population, because competitive swimmers undertake high training volumes, prolonged sessions and mixed aerobic–anaerobic workloads, so they represent a physiologically demanding model in which energy expenditure is substantial, repeated pool-based workouts continuously challenge fluid and electrolyte balance, and even subtle variations in nutritional intake may translate into measurable changes in performance, recovery and cellular hydration status [39,40,41].

## 2. Materials and Methods

### 2.1. Subjects

A total of thirty-nine competitive swimmers (mean age 19.7 ± 2.3 years; 22 males and 17 females) were enrolled and monitored over five months under standardized training conditions: the athletes followed training programs that were similar in intensity, duration, and frequency. All participants completed six weekly training sessions organized as follows: three sessions consisting of a dry-land pre-activation period followed by in-water training and a subsequent gym-based strength session, with a total duration of approximately 3 h; and three sessions consisting of a dry-land pre-activation period followed by in-water training, with a total duration of approximately 2 h. Athletes were recruited from a single competitive swimming academy and were approached during team meetings in collaboration with coaching staff. Participation was entirely voluntary and no financial or material incentives were provided. All participants met the following inclusion criteria: completion of six scheduled training sessions per week; absence of performance-enhancing drug use and of any medication; abstention from alcohol and caffeinated beverages for at least 15 h before testing; ability to complete an online questionnaire in Italian prior to the initial clinical evaluation; and willingness to provide written informed consent. Exclusion criteria included the presence of musculoskeletal injury. Data collection took place during the first half of the competitive season, and all assessments were performed in the morning between 9:00 and 11:00 AM. All participants completed the online pre-visit survey and electronically signed the consent form before enrollment. The study was approved by the Lazio Area 5 Territorial Ethics Committee (Approval Code: N.57/SR/23, Approval Date: 7 November 2023), in accordance with the Declaration of Helsinki and its subsequent amendments. Recruitment began in February 2025 and by July 2025, a total of 39 surveys had been recorded. The study is registered on ClinicalTrials.gov (NCT07315529).

### 2.2. Survey

Adherence to the Mediterranean dietary pattern was assessed using the MEDI-LITE questionnaire, validated in 2017, with scoring performed in accordance with the original methodology wherein higher scores indicate greater adherence [42]. The survey was administered online before the first clinical assessment and was accessible via any internet-enabled device It was conceived to be self-administered, anonymous, and required approximately 30–40 min for completion.

Following baseline dietary assessment, participants were stratified into three groups according to habitual legume consumption: a control group (<1 serving/week with no dietary modification), a moderate-intake group (≈2 servings/week increased to 3 servings/week), and a high-intake group (≈3–4 servings/week increased to 6 servings/week). Dietary adherence was monitored every four weeks by a qualified nutrition professional to ensure that the prescribed adjustment in legume intake represented the only dietary modification. Moreover, the MEDI-LITE questionnaire was administered to participants also at the end of the study to confirm legume intake frequency.

### 2.3. Body Composition

Anthropometric and body composition data were collected during the initial clinical assessment by trained personnel using standardized protocols. All measurements were taken in the morning, with participants dressed in light indoor clothing and barefoot. Body weight was recorded using balance-beam scale (Seca 700, Seca GmbH & Co. KG, Hamburg, Germany). Height was rounded to the closest 0.5 cm. BMI was calculated as weight divided by squared height in meters (kg/m^2^). Body composition was assessed while using a BIA phase-sensitive system by experienced observers (an 800-µA current at a frequency single-frequency of 50 kHz BIA 101, Akern Bioresearch, Florence, Italy), following a standardized pre-assessment protocol (no exercise, food, fluids, caffeine, or alcohol for 12 h before measurements). The electrodes were placed on the hand and the ipsilateral foot, according to Kushner [43]. After subjects were made to rest in the supine position for 5–10 min, measurements were taken while they were laying supine on a table with their arms ≥ 30 degrees away from their torso with legs separated. After hair removal and cleaning with alcohol, proximal electrodes should be placed on the right side of the body at the wrist (dorsal surface at the ulnar styloid process) and ankle (dorsal surface between the malleoli). After placing the proximal electrodes, distal electrodes can be placed at a distance of at least 5 cm between electrode centres or placed on the hand (dorsal surface of the metacarpal phalangeal joint, 1 cm proximal to the knuckle of the middle finger) and foot (dorsal surface of the metatarsal phalangeal joint, 1 cm proximal to the joint of the second toe). The room temperature during measurements was 22 °C. PhA is calculated as the relationship between the resistance (R) of tissues, which is mainly dependent on tissue hydration and their reactance (Xc), which is associated with cellularity, cell size, and integrity of the cell membrane, according to the following formula: PhA (°, degrees) = Xc/R × (180/π).

### 2.4. Autonomic and Functional Parameters

Autonomic parameters, including heart rate variability (HRV) and cardiac coherence were measured with Inner Balance Coherence Plus. The HRV assessment was performed in the morning immediately after waking up, before engaging in any physical activity. Measurements were obtained in the supine position, with the hands placed on the abdomen and the legs extended. Participants followed a controlled breathing pattern (10 s inhalation + 5 s exhalation), and HRV was recorded for a total duration of 5 min. Cardiac coherence was assessed using the Inner Balance Coherence Plus device (HeartMath Institute, Boulder, CO, USA), which provides a proprietary coherence score based on the regularity and spectral concentration of heart rate variability oscillations, reflecting autonomic synchronization. The critical swim speed test was performed in an indoor pool with a depth of 1.80 m, containing 6 lanes that were 25 m long and 2 m wide, with an average water temperature of 28 °C. After a standardized in-water warm-up, the test consisted of the following sequence: a 400-m maximal-speed swim starting from the water; 8 min of active recovery with continuous swimming; a 50-m maximal-speed swim starting from the water.

### 2.5. Gender Difference Evaluation

Given the well-documented sex-related differences in body composition, hydration status, and bioelectrical properties, as well as potential differences in autonomic regulation, an exploratory gender-specific analysis was conducted to assess whether the physiological responses to the dietary intervention differed between male and female athletes.

### 2.6. Statistical Analysis

Statistical analyses were conducted using IBM SPSS Statistics (version 27; IBM Corp., Armonk, NY, USA) and supplemented with Python scientific libraries (version 3.13; pandas, SciPy, statsmodels) to ensure robustness and reproducibility. Data quality checks included assessment of completeness and cleaning prior to analysis. Baseline between-group differences in continuous variables (age, weight, BMI, body composition parameters) were assessed using one-way analysis of variance (ANOVA) after verifying normality. Categorical variables (sex) were compared using χ^2^ tests. For longitudinal outcomes, within-group changes from baseline to end-of-study were evaluated using paired *t*-tests for normally distributed variables, or Wilcoxon signed-rank tests when distributional assumptions were not met. Between-group differences in longitudinal changes (Δ = end − baseline) were assessed using one-way ANOVA, followed by Tukey’s post-hoc correction for multiple comparisons. To account for potential confounding by sex, an analysis of covariance (ANCOVA) was additionally performed with sex as a covariate. All tests were two-sided, and a *p*-value < 0.05 was considered statistically significant.

## 3. Results

This prospective interventional study with a structured educational component purpose was completed by 39 subjects, who were divided into three groups, with 13 participants in each group. Adherence to the legume intake protocol was monitored monthly through nutritional interviews conducted by a qualified dietitian. Overall compliance was high: participants assigned to the 3-legume and 6-legume groups reported consumption consistent with their prescribed weekly portions, with no meaningful deviations observed during the intervention period. Participants across the three groups (control, 3-legumes and 6-legumes) were comparable at baseline in terms of age, sex distribution and anthropometric measures, with no statistically significant differences in body weight or BMI (Table 1). Body weight and BMI also remained stable throughout the intervention, with no between-group differences at follow-up (*p* = 0.159 and *p* = 0.704, respectively).

Table 1 presents the baseline demographic and anthropometric characteristics of the participants divided by intervention group (control, 3 servings of legumes, 6 servings of legumes). The data are expressed as mean ± standard deviation for continuous variables and as absolute frequencies and percentages for categorical variables. Comparisons between groups were performed using analysis of variance (ANOVA) or Chi-square (χ^2^) tests, depending on the nature of the variable. The variables reported include body mass index (BMI).

Regarding bioelectrical parameters, no significant difference in resistance (Rz) was observed in the control group (*p* = 0.219), in the 3-legumes group (*p* = 0.094), or in the 6-legumes group (*p* = 0.193). As for reactance (Xc), a significant reduction was observed in the control group (*p* = 0.029).

**Figure 1:**
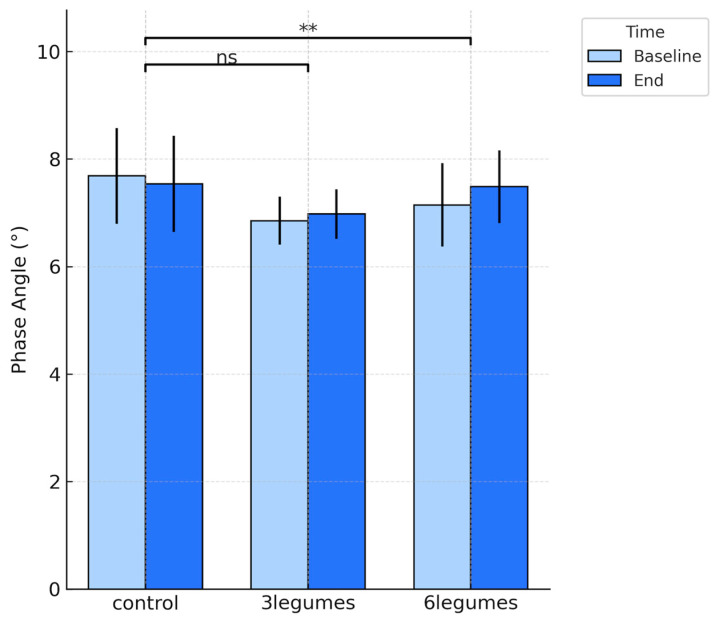
Changes in Phase Angle (degrees) at baseline and end of the intervention across the three study groups (control, 3-legumes, and 6-legumes). Bars represent mean ± SD for each time point. Significance markers above the plot denote between-group differences based on Tukey post-hoc comparisons applied to the change values (end − baseline). Asterisks denote the statistical significance of differences versus the control group ** *p* < 0.01. ns: not significant.

By contrast, a statistically significant increase in phase angle was detected exclusively in the 6-legumes group (Δ = + 0.34 ± 0.35°, *p* = 0.004, d = 0.97) (Figure 1), while values in the control and 3-legumes groups remained essentially unchanged (*p* = 0.255 and *p* = 0.161, respectively). Between-group analyses confirmed a significant effect of legume intake on phase angle change (*p* = 0.007).

Table 2 shows the bioimpedance and cardiac autonomic function parameters measured at baseline and at the end of the intervention, with the relative changes within each group. Values are reported as mean ± standard deviation. Intra-group changes were analysed using paired *t*-tests, while inter-group differences in changes were assessed using ANOVA and analysis of covariance (ANCOVA) adjusted for sex. Key abbreviations include ECW for extracellular water, ICW for intracellular water, Rz indicating electrical resistance related to total body water, Xc as a measure of reactance reflecting cell membrane capacity, and PhA (phase angle) summarising the state of cell integrity and body cell mass.

**Figure 2:**
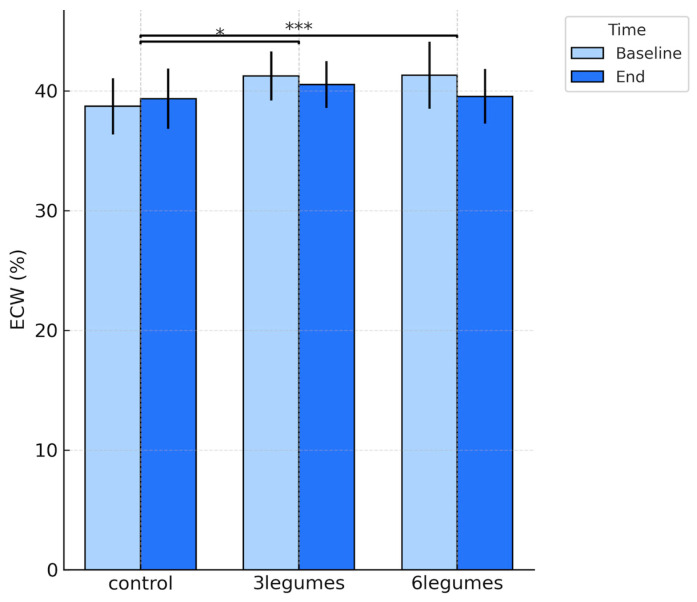
Extracellular Water (ECW) at baseline and end of the intervention in the three study groups. Mean ECW (%) ± SD is presented for the control, 3-legumes, and 6-legumes groups at baseline and at the end of the 5-month intervention. Bars in light blue represent baseline values, and dark blue bars represent end-of-study values. Significance lines above the bars indicate between-group comparisons based on the change in ECW (delta = end − baseline). Asterisks denote the statistical significance of differences versus the control group (* *p* < 0.05; *** *p* < 0.001).

**Figure 3:**
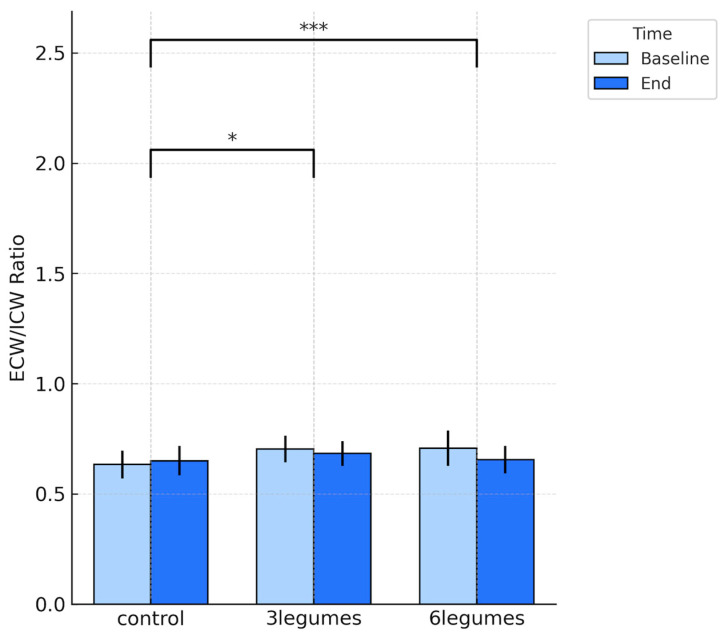
Extracellular-to-intracellular water ratio (ECW/ICW) at baseline and end of the study for the control, 3-legumes, and 6-legumes groups. Data are expressed as mean ± SD. Between-group significance for change over time (end − baseline) is displayed above the figure using Tukey-adjusted comparisons. Asterisks denote the statistical significance of differences versus the control group * *p* < 0.05, *** *p <* 0.001.

Water-distribution indices showed a coherent pattern across intervention groups. Both legume groups experienced a reduction in extracellular water (*p* = 0.047 and *p* < 0.001, respectively) and a corresponding increase in intracellular water, with the largest shifts observed in the 6-legumes group (*p* < 0.001), whereas the control group showed no meaningful change (*p* = 0.161) (Table 2, Figure 2 and Figure 3). As a result, the ECW/ICW ratio decreased significantly in the intervention groups (*p* = 0.049 in 3Legumes group and *p* < 0.001 in 6Legumes group) and remained stable in controls, indicating a more favourable redistribution of body water towards the intracellular compartment with higher legume intake (*p* = 0.154).

Autonomic parameters displayed similar dose–response trends. A significant increase in heart rate variability (HRV) was observed exclusively in the 6Legumes group (Δ = +6.92 ± 5.02, *p* = 0.0003, d = 1.38), with non-significant changes in the 3-legumes group and in the control group (*p* = 0.169 and 0.275, respectively). Similarly, cardiac coherence improved significantly only within the 6Legumes group (Δ = +0.40 ± 0.35, *p* = 0.0015, d = 1.14), with no significant changes found in the control or 3Legumes groups (*p*= 0.309 and *p* = 0.143, respectively). Critical swim speed showed a positive, albeit non-significant, trend in all group (*p* = 0.087).

Table 3 reports performance indicators, including Critical Swim Speed (CSS), and heart rate variability (HRV) parameters at baseline and after intervention, broken down by legume consumption groups. Data are expressed as mean ± standard deviation. CSS is a measure of swimming performance (m/s), while HRV reflects autonomic nervous system activity. Comparative analyses included paired *t*-tests within groups and ANOVA for differences between groups on variations, with Tukey’s post-hoc test for multiple comparisons. Legend: *p* < 0.05 = significant (*), *p* < 0.01 = **, *p* < 0.001 = ***.

Gender-specific analyses (Table 4) revealed a significantly greater increase in phase angle in females compared with males (*p* = 0.020), with a moderate effect size (r = 0.44). No statistically significant sex differences were observed for extracellular water, intracellular water, or the ECW/ICW ratio, although females showed trends toward greater reductions in ECW and ECW/ICW ratio and greater increases in ICW, with small-to-moderate effect sizes (d ≈ 0.42–0.48). Measures of autonomic function, including heart rate variability and cardiac coherence, did not differ significantly between sexes, despite a non-significant trend toward greater HRV improvement in females (r = 0.26). No significant sex differences were detected for resistance (Rz). Reactance (Xc) showed a non-significant trend toward greater improvement in females (*p* = 0.072), with a moderate effect size (d = −0.59).

Collectively, these analyses reinforce the main findings by indicating that increasing legume intake within a Mediterranean dietary framework is associated with graded improvements in fluid distribution and autonomic regulation beyond the effects of baseline status and sex.

## 4. Discussion

The primary objective of this study was to examine whether a higher intake of legumes—reflecting greater adherence to the Mediterranean Diet—was associated with favorable adaptations in body composition, bioelectrical, and autonomic parameters in competitive swimmers. The principal finding was a statistically significant increase in phase angle (PhA) observed in the 6Legumes group, suggesting improved cellular integrity and bioelectrical function following the intervention. This result is consistent with previous evidence identifying PhA as a reliable, non-invasive biomarker of cellular health that reflects both cell membrane integrity and functional status [44,45,46]. PhA is influenced by a range of physiological and nutritional factors, including age, sex, and dietary quality, and lower values have been associated with reduced muscle quality, diminished strength, and compromised nutritional or functional status [26]. Moreover, these findings align with emerging evidence from athletic populations linking plant-rich dietary patterns with improvements in bioelectrical parameters and performance markers [36,40,47]. A recent cross-sectional study of competitive athletes demonstrated that higher consumption of minimally processed plant foods—including legumes, nuts, and whole grains—was positively associated with phase angle values, independent of total energy intake and body composition [36]. In elite synchronized swimmers, bioelectrical impedance vector analysis revealed dynamic shifts in hydration status in response to high-volume training, confirming the sensitivity of ECW/ICW metrics to nutritional and physiological stressors in aquatic sports [40]. Regarding autonomic outcomes, observational data from physically active adults indicate that greater adherence to Mediterranean-style diets correlates with enhanced heart rate variability, potentially mediated by reduced systemic inflammation [48,49]. However, to our knowledge, no randomised intervention has specifically tested legume intake effects on BIA-derived cellular health markers or autonomic function in sport-specific cohorts such as competitive swimmers, highlighting the novelty of the present study.

Accordingly, the increase in PhA among participants with higher legume consumption may reflect enhanced membrane functionality and metabolic efficiency, potentially driven by the nutrient density and antioxidant properties characteristic of a plant-rich Mediterranean dietary pattern.

The observed associations may be mediated by legume-derived nutrients with established biological effects. Soluble fibre and resistant starch from pulses can modulate gut microbiota composition and short-chain fatty acid production, which in turn influence systemic inflammation, gut barrier integrity and fluid homeostasis [50,51,52]. Polyphenols naturally present in legumes exert antioxidant and anti-inflammatory actions that may help preserve cell membrane integrity and limit oxidative damage to bioelectrical properties [53,54]. In addition, the high potassium and magnesium content of Mediterranean-style plant foods, including legumes, supports intracellular electrolyte balance and membrane potential stability, while an adequate combination of plant protein sources within a Mediterranean dietary pattern can provide sufficient indispensable amino acids to meet athletic requirements without impairing muscle function [15,55]. Enhanced vagal modulation may therefore reflect the combined effects of reduced low-grade inflammation, improved glycaemic control and favourable shifts in microbiota-derived metabolites, although these pathways remain speculative in the present context [55,56,57]. Importantly, our study did not assess inflammatory biomarkers, oxidative stress indices, amino acid profiles or gut microbiota composition, and the proposed mechanisms should thus be regarded as hypothesis and require confirmation in dedicated mechanistic trials.

In support of this interpretation, Barrea et al. reported that greater adherence to the Mediterranean Diet was positively correlated with PhA, reinforcing the role of this dietary model in promoting cellular integrity and overall physiological well-being [58]. The consistent directionality between our findings and existing evidence supports the notion that plant-based dietary components—particularly legumes—can beneficially modulate cellular and functional parameters, even in athletic populations typically characterized by high energy expenditure and elevated protein requirements.

Beyond bioelectrical parameters, increasing legume intake over five months was associated with reductions in extracellular water (ECW), increases in intracellular water (ICW), and improvements in cardiac coherence among young competitive swimmers, with the largest changes observed in the 6-legumes/week group and smaller, directionally consistent shifts in the moderate-intake group. These patterns—lower ECW, higher ICW, reduced ECW/ICW ratio, and higher cardiac coherence—may reflect more favourable fluid compartment regulation and parasympathetic modulation, consistent with evidence that plant-rich dietary patterns influence autonomic function through metabolic and inflammatory pathways [48,59,60]. However, given the modest sample size and near-significant between-group differences for HRV and coherence, these findings should be interpreted cautiously as hypothesis-generating rather than definitive.

Although CSS did not show statistically significant changes across groups, both legume intervention arms exhibited positive trends (+0.010–0.011 m/s) compared to a small decline in controls. This lack of significance may reflect the relatively short five-month intervention duration, the subtlety of dietary effects on peak performance metrics, or the limited sensitivity of CSS to nutritional interventions in already well-trained athletes. Notably, the study was not formally powered to detect performance differences, which require larger effect sizes and sample sizes than the cellular hydration and autonomic outcomes that served as primary endpoints.

Strengths of the study include the controlled longitudinal design with repeated bioelectrical and autonomic assessments over five months under standardised training conditions, minimising confounding from training variations. Validated tools were employed throughout: the MEDI-LITE questionnaire for Mediterranean diet adherence, phase-sensitive BIA for cellular hydration metrics, Inner Balance Coherence Plus for HRV and cardiac coherence, and the critical swim speed test as a sport-specific performance measure. Rigorous dietary monitoring ensured legume intake was the sole targeted modification, while statistical analyses adjusted for sex as a key confounder. These methodological features enhance the internal validity of the observed associations between legume consumption and physiological outcomes in competitive swimmers.

Despite the promising observations, several limitations must be acknowledged. First, the modest sample size (*n* = 39) may have limited statistical power, particularly for outcomes with high inter-individual variability such as heart rate variability and critical swim speed. In addition, dietary adherence relied on self-reported compliance verified bi-weekly by nutritionists, rather than objective tools such as food diaries, weighed intake records or urinary biomarkers, which may have introduced reporting bias despite careful monitoring. Moreover, although conducted under ‘standardised training conditions’, the study did not objectively quantify training load variations (e.g., via session-RPE, heart rate metrics or GPS data), and potential differences in coach-led programming or seasonal progression across the five-month period cannot be fully excluded. Fourth, while sex was included as a covariate in primary analyses, females showed greater improvements in phase angle (*p* = 0.020), suggesting possible sex-specific responses that warrant exploration in larger, stratified trials. Finally, recruitment from a single sports centre limits generalisability, and the intervention duration may have been insufficient to capture peak performance adaptations. These limitations underscore the preliminary nature of the findings and the need for larger, multi-centre randomised controlled trials with comprehensive objective assessments.

Future studies with larger samples and longer durations are needed to confirm and expand on these findings.

The data presented in this study allow us to conclude that increasing legume intake to 6 servings per week within a Mediterranean dietary framework appears feasible for competitive swimmers and is associated with favourable shifts in cellular hydration parameters and autonomic markers under standardised training conditions. These preliminary observations suggest that legume-rich plant proteins may represent a practical, nutrient-dense option to complement traditional sports nutrition strategies, potentially supporting physiological adaptations relevant to training and recovery. While larger randomised trials are needed to confirm these associations and explore performance implications, athletes and coaches may consider incorporating higher legume consumption as part of a balanced Mediterranean-style eating pattern.

## 5. Conclusions

Higher adherence to the Mediterranean diet, achieved through increased legume intake (particularly 6 servings/week) was associated with significant improvements in bioelectrical and hydration parameters. Specifically, these included increased phase angle, elevated intracellular water, reduced extracellular water, and a decreased ECW/ICW ratio, collectively indicating enhanced cellular integrity and optimized fluid distribution. These findings suggest that greater adherence to a plant-rich Mediterranean dietary pattern exerts beneficial effects on cellular function, hydration status, and autonomic nervous system performance in competitive swimmers. Increased legume consumption within a Mediterranean dietary framework was associated with enhanced cellular integrity, favorable shifts in body water distribution, and improved autonomic regulation in competitive swimmers—physiological adaptations consistent with greater overall efficiency.

Further interventional studies involving larger athlete cohorts are warranted to confirm these observations and elucidate the underlying mechanistic pathways. From a practical perspective, these results indicate that coaches and nutritionists may consider promoting a legume-enriched Mediterranean dietary approach, as the intake of up to six servings of legumes per week is a feasible and sustainable strategy that can be readily integrated into athletes’ habitual meal plans to support physiological efficiency and training adaptation.

Future research should also explore potential mechanistic pathways linking higher legume consumption to the observed physiological adaptations, including inflammatory markers, gut microbiota composition and function, amino acid metabolism, and autonomic biomarkers such as heart rate variability and cardiac coherence indices.

Taken together, our findings support the relevance of a legume-enriched Mediterranean dietary pattern as a feasible, health-promoting strategy that may contribute to optimising both physiological resilience and performance in athletes.

## Figures and Tables

**Table 1:** Baseline demographic and anthropometric characteristics of participants divided by intervention group (control, 3 servings of legumes, 6 servings of legumes).

	Control Group	3Legumes Group	6Legumes Group	*p*
Age (mean ± SD)	20.46 ± 2.93	18.38 ± 2.1	20.38 ± 2.66	0.082
Weight (mean ± SD)	68.52 ± 13.38	61.11 ± 11.06	68.28 ± 7.49	0.159
BMI (mean ± SD)	21.63 ± 2.25	21.03 ± 2.57	21.75 ± 2.19	0.704
Gender (F/M)				0.482
Female (*n*/%)	4 (30.7)	6 (46.2)	7 (53.8)	
Male (*n*/%)	9 (69.23)	7 (53.8)	6 (46.2)	

**Table 2:** Bioelectrical and autonomic parameters at baseline and at the end of the study, with variations observed in each intervention group.

	Variable	Baseline	End of the Study	*p*
Control group	Rz	470.92 ± 88.47	460.92 ± 68.48	0.219
	Xc	62.38 ± 4.96	59.46 ± 5.95	0.029
	Phase Angle	7.68 ± 0.89	7.54 ± 0.90	0.255
	ECW	38.70 ± 2.35	39.33 ± 2.53	0.161
	ICW	61.30 ± 2.35	60.67 ± 2.53	0.161
	ECW/ICW ratio	0.63 ± 0.06	0.65 ± 0.07	0.154
	HRV	69.69 ± 8.36	68.23 ± 9.67	0.275
	Average Coherence	3.30 ± 1.37	2.97 ± 1.32	0.309
3Legumes group	Rz	508.00 ± 69.25	495.23 ± 67.00	0.094
	Xc	58.46 ± 6.13	58.15 ± 8.03	0.803
	Phase Angle	6.85 ± 0.44	6.98 ± 0.46	0.161
	ECW	41.23 ± 2.06	40.53 ± 1.96	0.047
	ICW	58.77 ± 2.06	59.47 ± 1.96	0.047
	ECW/ICW ratio	0.70 ± 0.06	0.68 ± 0.06	0.049
	HRV	70.62 ± 8.74	76.69 ± 11.87	0.169
	Average Coherence	2.36 ± 0.79	2.65 ± 0.77	0.143
6Legumes group	Rz	487.46 ± 86.23	473.69 ± 86.91	0.193
	Xc	59.85 ± 6.43	61.38 ± 7.22	0.328
	Phase Angle	7.15 ± 0.78	7.48 ± 0.68	0.004
	ECW	41.30 ± 2.81	39.53 ± 2.28	<0.001
	ICW	58.70 ± 2.81	60.47 ± 2.28	<0.001
	ECW/ICW ratio	0.71 ± 0.08	0.66 ± 0.06	<0.001
	HRV	69.77 ± 5.17	76.69 ± 4.71	<0.001
	Average Coherence	2.45 ± 0.77	2.85 ± 0.80	0.001

**Table 3:** Summary of Physiological, Autonomic, and Performance Parameters.

Parameter	Control Δ ± SD	3Legumes Δ ± SD	6Legumes Δ ± SD	*p* (Intra, 6Legumes)	ANOVA *p* (Between Groups)
Resistance (Rz)	−10.00 ± 27.81	−12.77 ± 25.33	−13.77 ± 35.99	0.193	0.947
Phase Angle (°)	−0.15 ± 0.44	+0.12 ± 0.30	+0.34 ± 0.35	<0.01	0.007
Extracellular Water (%)	+0.63 ± 1.52	−0.70 ± 1.14	−1.77 ± 0.93	<0.001	<0.0001
Intracellular Water (%)	−0.63 ± 1.52	+0.70 ± 1.14	+1.77 ± 0.93	<0.001	<0.0001
ECW/ICW Ratio	+0.020 ± 0.040	−0.020 ± 0.034	−0.051 ± 0.028	<0.001	<0.0001
Heart Rate Variability (HRV)	−1.46 ± 4.61	+6.08 ± 14.99	+6.92 ± 5.02	0.0003	0.059
Cardiac Coherence (CC)	−0.33 ± 1.12	+0.21 ± 0.66	+0.40 ± 0.35	0.0015	0.059
Critical Swim Speed (CSS, m/s)	−0.0034 ± 0.0158	+0.0101 ± 0.0183	+0.0107 ± 0.0192	0.067	0.087

**Table 4:** Comparison Between Males and Females (Δ end − baseline).

Parameter (Δ)	Male Mean ± SD (*n*)	Female Mean ± SD (*n*)	Test	*p*-Value	Effect Size
Phase Angle	−0.036 ± 0.444 (22)	+0.288 ± 0.280 (17)	Mann–Whitney U	0.020	r = 0.44
Extracellular Water (ECW)	−0.323 ± 1.692 (22)	−0.988 ± 1.299 (17)	Welch *t*-test	0.173	d = 0.42
Intracellular Water (ICW)	+0.323 ± 1.692 (22)	+0.988 ± 1.299 (17)	Welch *t*-test	0.173	d = −0.42
ECW/ICW Ratio	−0.009 ± 0.047 (22)	−0.030 ± 0.038 (17)	Welch *t*-test	0.124	d = 0.48
HRV	+2.00 ± 7.89 (22)	+6.24 ± 12.06 (17)	Mann–Whitney U	0.173	r = 0.26
Cardiac Coherence	+0.159 ± 0.942 (22)	+0.065 ± 0.665 (17)	Mann–Whitney U	0.712	r = −0.07
Resistance (Rz)	−9.46 ± 23.69 (22)	−15.71 ± 35.76 (17)	Welch *t*-test	0.539	d = 0.21
Reactance (Xc)	−1.82 ± 4.81 (22)	+1.06 ± 4.80 (17)	Welch *t*-test	0.072	d = −0.59

Comparison of mean changes (Δ = post − baseline) between male and female athletes across functional, bioelectrical, and autonomic parameters. Welch’s *t*-test was applied when both groups were normally distributed; otherwise, Mann–Whitney U test was used. Effect sizes are reported as Cohen’s d (parametric) or rank-biserial r (non-parametric).

## Data Availability

The raw data supporting the conclusions of this article will be made available by the authors on request.
